# Intraocular pressure measurement and association with corneal biomechanics in patients underwent Descemet’s stripping with endothelial keratoplasty: a comparative study

**DOI:** 10.3389/fmed.2024.1384694

**Published:** 2024-07-12

**Authors:** Huiyu Chen, Suqian Wu, Lijia Tian, Yue Li, Jiaxu Hong, Yulan Wang, Jianjiang Xu

**Affiliations:** ^1^Shanghai Eye Diseases Prevention & Treatment Center/Shanghai Eye Hospital, School of Medicine, Tongji University, Shanghai, China; ^2^National Clinical Research Center for Eye Diseases, Shanghai Engineering Research Center of Precise Diagnosis and Treatment of Eye Diseases, Shanghai, China; ^3^Eye Institute and Department of Ophthalmology, Eye & ENT Hospital, Fudan University, Shanghai, China; ^4^NHC Key Laboratory of Myopia and Related Eye Diseases, Chinese Academy of Medical Science, Shanghai, China; ^5^Shanghai Key Laboratory of Visual Impairment and Restoration, Shanghai, China

**Keywords:** corneal biomechanics, DSEK, intraocular pressure, Corvis ST, tonometer

## Abstract

**Purpose:**

To compare corneal biomechanical properties and intraocular pressure (IOP) measurements in patients who underwent Descemet’s stripping with endothelial keratoplasty (DSEK) with those of the follow healthy eyes.

**Methods:**

In this retrospective comparative study, a total of 35 eyes of 35 patients who underwent DSEK by a single surgeon from 2015.02 to 2019.12 were enrolled along with their fellow healthy eyes. Corneal biomechanical parameters were assessed at least 3 months post-DSEK using Corneal Visualization Scheimpflug Technology (CST). IOP was measured by CST, Goldmann applanation tonometry (GAT), and MacKay-Marg tonometer.

**Results:**

Central corneal thickness (CCT) and stiffness parameter at first applanation (SP-A1) were significantly increased after DSEK when compared to the fellow eyes. In DSEK eyes, biomechanically-corrected intraocular pressure (bIOP) and MacKay-Marg IOP correlated significantly with GAT IOP measurements, with bIOP showed the lowest IOP values. All the IOP values did not correlate with CCT. However, GAT-IOP and MacKay-Marg IOP showed a positive correlation with SP-A1.

**Conclusion:**

The corneal stiffness increased after DSEK. Central corneal thickness may have less influence than corneal biomechanics on IOP measurements in eyes after DSEK. Biomechanically-corrected IOP obtained by CST seemed to be lower than other tonometry techniques in DSEK eyes, perhaps because of correction for corneal stiffness, CCT and age.

## 1 Introduction

Over the last decade, Descemet’s stripping with endothelial keratoplasty (DSEK), a selective replacement of the diseased corneal endothelium, has become the most commonly performed procedure for treating corneal endothelial dysfunction (1). This technique surpass penetrating keratoplasty in terms of rapid visual recovery, preserved corneal sensation, tectonic stability, and absence of suture-related complications (1, 2). Post-DSEK intraocular pressure (IOP) elevation, one of the most common complications, accelerates primary graft failure (3). The reported incidence ranges from 16.7 to 54%, mostly due to cumulative use of corticosteroids (4–10). Therefore, as with all types of keratoplasty, IOP monitoring after DSEK remains essential.

However, an accurate measurement of IOP after DSEK remains challenging. For example, Goldmann applanation tonometer (GAT) is considered as the gold standard for IOP measurement, but a growing body of research suggests that it is inevitably affected by corneal biomechanics and central corneal thickness (CCT) (11, 12), which is affected by the additional donor graft in DSEK (13, 14). Tonopen is a hand-held electronic tonometer based on the MacKay-Marg principle, but flattens the cornea in a smaller area compared to GAT, thus reducing the difference between the flattening pressure and the real IOP (15). It has been reported that Tono-Pen XL was less affected by CCT in eyes that underwent penetrating keratoplasty (16).

Corneal biomechanics is the study of corneal deformation in response to external forces. It can be analyzed *in vivo* with both the Ocular Response Analyzer (ORA) and the Corvis ST Tonometer (CST). In particular, CST can provide assessments of specific changes in the corneal elastic properties based on its ultra-high-speed Scheimpflug technology (17). It has been reported that CST is more sensitive to corneal biomechanical changes after cataract surgery than the ORA (18, 19). Recently, biomechanically-corrected intraocular pressure (bIOP), a newly released CST parameter, was corrected for corneal stiffness, CCT and age (20).

The purpose of our study was to investigate the effects of DSEK on corneal biomechanics via CST and to explore the relationship between corneal biomechanics and IOP obtained by CST, GAT, and MacKay-Marg tonometer.

## 2 Materials and methods

### 2.1 Patients

This retrospective comparative study included 35 patients aged 18–85 years old, who underwent DSEK for the treatment of corneal endothelial decompensation and had healthy contralateral eyes by one surgeon at the Eye & ENT Hospital of Fudan University (Shanghai, China). Exclusion criteria for the post-DSEK eyes were as follows: (1) operative eyes had corneal stromal layer surgery history or trauma, (2) graft detachment with air injection, (3) persistent epithelial defect, (4) graft failure or endothelial immunologic rejection within 3 months postoperative, (5) operative eyes undergone any other ocular surgery during follow-up period. Exclusion criteria for the healthy fellow eyes were corneal abnormalities such as guttae, edema, scars; glaucoma; history of ocular surgery or other ocular abnormalities. This study was approved by the Ethics Committee of the Eye & ENT Hospital of Fudan University (Approval No. 2015020) and adhered to the tenets of the Declaration of Helsinki.

### 2.2 Assessments

All the participants underwent a routine examination including slit lamp biomicroscopy and best corrected visual acuity to check the exclusion criteria. Corneal bio-mechanical properties were assessed by a single investigator using the Corvis ST (CST; Oculus, Wetzlar, Germany). As previously described, this instrument releases a rapid air puff of air onto the cornea and captures the entire corneal deformation process using an ultra-high-speed Scheimpflug camera (17). The corneal response is divided into an inward applanation (flattening), deformation to maximum concavity, and the second outward applanation as the cornea returns to its original shape. The recorded images were analyzed via the built-in CST software (ver. 1.3r1538).

In addition to the CST test, IOP was also measured by GAT and MacKay-Marg tonometer (TonoPen AVIA; Reichert Inc., Buffalo, New York) according to manufacturers’ instructions. For MacKay-Marg tonometer measurements, only values with a coefficient of variation of 5% or less were accepted. A total of three consecutive measurements were obtained and averaged for each patient.

### 2.3 Statistical analyses

Statistical analyses were performed using SPSS 22.0 software. All statistics were presented as mean ± standard deviation. The Kolmogorov–Smirnov test was used to assess the normality of the data. If the data were normally distributed, a two-tailed paired Student’s *t*-test with Bonferroni correction was used for multiple comparisons, otherwise a Wilcoxon signed rank test was used for the statistical analysis with the contralateral eye as the control. One way ANOVA with Bonferroni correction was used to compare the differences between three IOP measurements. Bland–Altman analysis was performed to demonstrate the agreement between the three IOP measurements. The correlation between IOP and corneal biomechanical parameters was analyzed by Pearson’s correlation coefficient. A *p* < 0.05 was considered statistically significant.

## 3 Results

As shown in Table 1, a total of 35 patients, 14 males and 21 females, who underwent DSEK in one eye were enrolled in this study, with a mean age of 55.7 ± 14.8 years (range, 24–82 years). The mean followed-up was 9.3 ± 8.4 months (3–30 months). The initial causes of corneal endothelial decompensation were peudophakic bullous keratopathy (*n* = 22), iridocorneal endothelial syndrome (*n* = 7), bullous keratopathy after phakic IOL insertion (*n* = 5), and herpes simplex virus corneal endotheliitis (*n* = 1). All enrolled subjects maintained clear corneas, insignificant edema, and no graft failure during follow-up.

**Table 1:** Demographic characteristics of the study samples (*n* = 35).

	Mean	SD	Range
Age (year)	55.6	14.8	24–82
Follow-up time (month)	9.3	8.4	3–30
Sex: male, *n* (%)	14 (40%)		
Eye side: right, *n* (%)	14 (40%)		
The causes of endothelial decompensation, *n*			
Pseudophakic bullous keratopathy	22		
Iridocorneal endothelial syndrome	7		
Bullous keratopathy after phakic IOL insertion	5		
Herpes simplex virus corneal endotheliitis	1		

Corneal biomechanical results are summarized in Table 2. Overall, statistical differences were observed between the two groups in applanation velocity (A1V; *p* = 0.004) and stiffness parameter at first applanation (SP-A1) (*p* < 0.0001). The SP-A1 is a novel indicator for corneal stiffness (21). Significantly higher SP-A1 values were found in operated eyes compared to the contra-lateral eyes (149.84 ± 29.22 mmHg/mm vs. 125.4 ± 20.18 mmHg/mm). CCT was significantly increased in operated eyes compared to normal eyes (660.5 ± 99.81 μm vs. 565.56 ± 57.78 μm, *p* < 0.0001). A slight but not significant decrease in peak distance (PD) and an increase in integrated radius (IR) were observed. These results indicate an increased corneal stiffness after DSEK.

**Table 2:** Corneal biomechanical parameters and different intraocular pressure measurements in post-DSEK eyes and contralateral healthy eyes.

Parameter	Post-DSEK eyes		Fellow eyes		*p*-value
	Mean	SD	Mean	SD	
A1T (msec)	7.19	0.59	7.04	0.39	**0.050** ^**#**^
A1L (mm)	2.27	0.33	2.27	0.31	0.960^*^
A1V (m/s)	0.13	0.24	0.14	0.18	**0.004** ^*^
A2T (msec)	21.20	1.18	21.27	0.56	**0.050** ^**#**^
A2L (mm)	1.80	0.53	1.90	0.48	0.641^#^
A2V (m/s)	−0.24	0.13	−0.27	0.06	0.106^#^
HCT (msec)	16.97	1.66	16.49	2.09	0.870^#^
HCDA (mm)	1.11	0.29	1.06	0.15	0.878^#^
PD (mm)	4.87	0.52	4.91	0.47	0.725^*^
HCR (mm)	6.27	1.26	6.45	1.34	0.422^#^
SP-A1	149.84	29.22	125.40	20.18	**<0.0001** ^*^
DAR	4.44	0.63	4.41	0.67	0.829^*^
IR (ms × mm^−1^)	9.08	2.13	8.85	1.53	0.537^*^
CCT (μm)	660.50	99.81	565.56	57.78	**<0.0001** ^*^
GAT-IOP (mmHg)	16.48	4.88	15.76	3.16	0.332^*^
MacKay-Marg IOP (mmHg)	15.53	4.43	15.27	3.56	0.683^*^
bIOP (mmHg)	14.09	4.96	14.41	3.71	0.698^*^

A1T/A2T, first/s applanation time; A1L/A2L, first/s applanation length; A1V/A2V, first/s applanation velocity; HCT, highest concavity time; HCDA, highest concavity deformation amplitude; PD, peak distance; HCR, highest concavity radius; SP-A1, stiffness parameter at first applanation; DAR, deformation amplitude ratio; IR, integrated radius; CCT, central corneal thickness; GAT, Goldmann applanation tonometer; IOP, intraocular pressure; bIOP, biomechanically-corrected intraocular pressure. ^*^p-values from paired *t*-test with Bonferroni correction; ^#^*p*-values from Wilcoxon signed rank test. Bold values mean ≤ 0.05.

**Figure 1:**
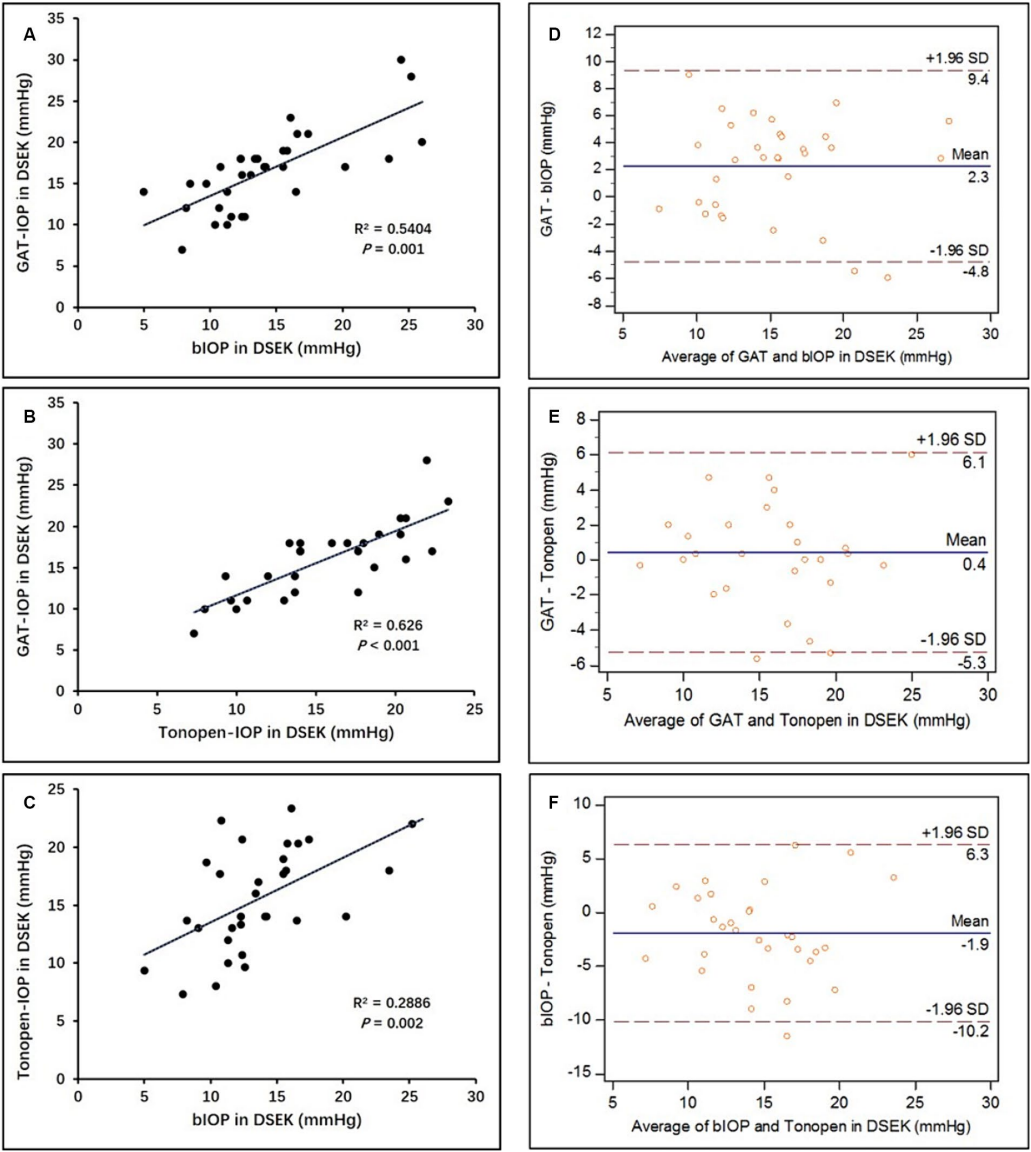
Correlation and agreement between the different tonometry methods in post-DSEK eyes. **(A–C)** Scatter graphs show the correlations of intraocular pressure measurements obtained by Corvis ST, Goldmann applanation, and MacKay-Marg tonometer (Tonopen AVIA), respectively. **(D–F)** Bland–Altman plots show the agreement of intraocular pressure measurements obtained by Corvis ST, Goldmann applanation, and MacKay-Marg tonometer, respectively.

The results of the IOP measurements are summarized in Table 2 with no statistical differences between the operated eyes and the contralateral eyes. All IOP values in the operated eyes measured by Corvis ST, GAT, and Tonopen AVIA were positively correlated with each other (Figures 1A–C). In post-DSEK eyes, bIOP showed the lowest IOP, while GAT IOP and MacKay-Marg IOP were similar as shown in Table 3. Bland–Altman analysis showed the 95% limits of agreement between these three tonometers in the post-DSEK eye group (Figures 1D–F). The range of MacKay-Marg—Corvis ST difference (95% LoA, −10.2 to +6.3 mmHg) was the highest, followed by GAT-Corvis ST difference (95% LoA, −4.8 to +9.4 mmHg).

**Table 3:** Test of differences between average intraocular pressure measurements in post-DSEK eyes.

Pair	IOP Difference (mmHg)	*p*-value
GAT—Corvis ST	2.29	**0.001**
GAT—MacKay-Marg	0.44	0.426
MacKay-Marg—Corvis ST	1.94	**0.015**

GAT, Goldmann applanation tonometer; IOP, intraocular pressure; bIOP, biomechanically-corrected intraocular pressure. *p*-values from one way ANOVA with Bonferroni correction for multiple comparisons. Bold values mean ≤ 0.05.

As shown in Table 4, correlation analyses were performed between the corneal bio-mechanical parameters and the IOP values of the post-DSEK eyes measured by Corvis ST, GAT, and MacKay-Marg tonometer. Most of the biomechanical parameters were strongly correlated with IOPs obtained from the three tonometry techniques. SP-A1 was positively correlated to GAT-IOP and MacKay-Marg IOP, whereas bIOP was not affected by SP-A1, suggesting that corneal stiffness had no impact on bIOP measurement. Of note, no significant correlation was found between CCT and any of these tonometers.

**Table 4:** Association between Corneal Biomechanical Parameters and different Intraocular Pressure measurements in post-DSEK eyes.

	GAT-IOP		bIOP		MacKay-Marg IOP	
	*r*	*p*	*r*	*p*	*r*	*p*
CCT (μm)	0.319	0.07	0.094	0.59	0.318	0.081
A1T (msec)	0.797	**<0.0001** ^*^	0.906	**<0.0001** ^*^	0.655	**<0.0001** ^*^
A1L (mm)	0.221	0.217	0.171	0.327	0.145	0.438
A1V (m/s)	−0.691	**<0.0001** ^*^	−0.739	**<0.0001** ^*^	−0.666	**<0.0001** ^*^
A2T (msec)	−0.448	**0.009** ^*^	−0.549	**0.001** ^*^	−0.612	**<0.0001** ^*^
A2L (mm)	0.535	**0.001** ^*^	0.395	**0.019** ^*^	0.445	**0.012** ^*^
A2V (m/s)	0.304	0.086	0.208	0.23	0.254	0.168
HCT (msec)	−0.044	0.809	−0.185	0.287	0.035	0.851
HCDA (mm)	−0.570	**0.001** ^*^	−0.665	**<0.0001** ^*^	−0.600	**<0.0001** ^*^
PD (mm)	−0.785	**<0.0001** ^*^	−0.678	**<0.0001** ^*^	−0.717	**<0.0001** ^*^
HCR (mm)	0.240	0.179	0.386	**0.022** ^*^	0.160	0.389
SP-A1	0.448	**0.009** ^*^	0.272	0.113	0.445	**0.012** ^*^
DAR	−0.640	**<0.0001** ^*^	−0.489	**0.003** ^*^	−0.506	**0.004** ^*^
IR (ms × mm^−1^)	−0.586	**<0.0001** ^*^	−0.614	**<0.0001** ^*^	−0.648	**<0.0001** ^*^

A1T/A2T, first/s applanation time; A1L/A2L, first/s applanation length; A1V/A2V, first/s applanation velocity; HCT, highest concavity time; HCDA, highest concavity deformation amplitude; PD, peak distance; HCR, highest concavity radius; SP-A1, stiffness parameter at first applanation; DAR, deformation amplitude ratio; IR, integrated radius; CCT, central corneal thick-ness; GAT, Goldmann applanation tonometer; IOP, intraocular pressure; bIOP, biomechanically-corrected intraocular pressure. *r* values from Pearson Correlation coefficient, 0.2 < *r* < 0.4 indicates weak correlation; 0.4 < *r* < 0.6 indicates middle correlation; *r* > 0.6 indicates strong correlation. +A positive correlation; −A negative correlation. ^*^Statistically significant. Bold values mean ≤ 0.05.

## 4 Discussion

Adequate management of IOP elevation after DSEK requires accurate measurement of intraocular pressure. Understanding the changes in corneal biomechanics after DSEK will help to better understand IOP measurement. In this study, we investigated the effects of DSEK surgery on corneal biomechanics using Corvis ST. As expected, CCT was significantly increased after DSEK compared to the fellow eyes. Significant enhancement of other CST parameters including A1T, A1V, A2T, and SP-A1 were also observed. In addition, we compared the newly released CST parameter, bIOP, with IOP values measured by two traditional and widely used tonometers, GAT and MacKay-Marg tonometer (Tonopen AVIA). The IOP measured by CST showed the lowest value compared to GAT and MacKay-Marg both in post-DSEK eyes and in fellow healthy eyes. In the light of some previous works that have investigated post-DSEK IOP measurement using techniques such as GAT and Tono-Pen (22), this study, to our knowledge, is the first approach to measure post-DSEK IOP using CST and to analyze the association of corneal biomechanical properties with IOP measurement after DSEK.

The new parameter SP-A1 was introduced to represent corneal stiffness, which was believed to have no correlation with corneal volume and age, and may reflect the changes of corneal stiffness closer to the real value (21). Corneas with higher ocular stiffness will have a higher value of SP-A1 (21). In this study, SP-A1 was significantly increased after DSEK, indicating the increased corneal stiffness. One potential explanation is that the transplanted cornea with additional donor grafts was significantly thicker than that of normal eyes, which required greater flattening pressure to induce corneal flattening reaction corresponding to prolonged first applanation time (A1T), reduced first applanation velocity (A1V), and shortened second applanation time (A2T) during corneal rebound. Although a corneal endodermis contributes little to corneal biomechanical properties, it is critical to keep the stroma from edema. As a hydrated tissue, the water content of the cornea has a critical effect on its biomechanical properties including tissue stiffness. Previous *in vitro* studies using unconfined compression tests and uniaxial tensile experiments have found corneal hydration level to be negative contributor to corneal tensile and compressive stiffness (23, 24). Meanwhile, SP-A1 was believed negatively correlated with corneal curvature, which was often decreased after DSEK due to grafts (25, 26). Some other variables may also be associated with SP-A1. For instance, an increase in IR and a decrease in PD may be responsible for the increase in corneal stiffness. In this study, post-DSEK PD and IR were found minimally decreased and increased without significant difference, respectively, suggesting that these variables may be less sensitive to corneal biomechanical measurements after DSEK compared to SP-A1. In the older version of CST, the highest concavity deformation amplitude (HCDA) and highest concavity radius (HCR) were obtained to analysis the ocular rigidity. High HCDA value and low HCR were associated with low ocular rigidity, corresponding to prolonged A1T, decreased A1V, and shortened A2T (27). However, no statistical difference in HCDA and HCR was found between post-DSEK eyes and the contralateral eyes in this study, which was in consistent with the previous results from Maeda et al. (28), indicating that SP-A1 may be a more sensitive parameter to reflect corneal stiffness than HCDA in DSEK eyes.

Previous studies found that eyes after DSEK had lower stiffness than normal eyes, including corneal hysteresis and corneal resistance factor assessed by ORA (13). However, Faramarzi et al. (14) showed the same conclusion as we did, that corneal biomechanics after DSEK were significantly increased and were comparable to the fellow healthy eyes using ORA. These conflicting results may be explained by differences in patient characteristics, graft thickness, and corneal edema status. It would also be necessary to further investigate the relationship between ORA and CST measurements applied to eyes after keratoplasty.

To determine the validity and reliability of the newly released parameter, bIOP obtained by CST in routine clinical practice, IOP measurements provided by this and two other widely used tonometry devices, GAT and MacKay-Marg tonometer (TonoPen AVIA), were recorded and compared in this study. Although the three devices showed high agreement, bIOP-CST showed an underestimated tendency in post-DSEK eyes compared to GAT (−2.3 mmHg, *p* = 0.001) and MacKay-Marg tonometer (−1.9 mmHg, *p* = 0.015). Further agreement analysis using Bland–Altman plots also revealed negative agreement between CST and the other two tonometers, suggesting that 70% of post DSEK eyes and 30% of healthy fellow eyes showed underestimated IOP difference between tonometers of more than ±3 mmHg. Thus, bIOP measurement by CST may not be interchangeable with GAT or MacKay-Marg tonometer. In agreement with our results, Karmiris et al. (29) reported smaller bIOP-CST values than GAT-IOP values in 113 adults. Similarly, Matsuura et al. (17), Hong et al. (30), and Vinciguerra et al. (31) reported underestimated bIOP values compared with GAT-IOP in patients with glaucoma. Considering that GAT-IOP is usually overestimated due to corneal edema and stiffness change after DSEK, we speculate that CST-IOP may be closer to the actual IOP value than GAT. On the other hand, several studies demonstrated conflicting evidence that CST tended to overestimate IOP values compared to those obtained by GAT (32–36).

Tonopen is a versatile tonometer with potential advantages in assessing IOP in the presence of corneal scarring or edema. Chang et al. (37) and Ohana et al. (38) reported that Tonopen XL is a reliable tool for measuring IOP after DSEK. Tonopen AVIA is a new hand-held flattening tonometer with higher sensitivity than the Tonopen XL. To our knowledge, the present study is the first report to measure post-DSEK IOP using Tonopen AVIA. Bland–Altman plots showed high agreement between Tonopen AVIA and GAT with a mean difference of 0.4 mmHg in post-DSEK eyes. The flattening range of the Tonopen AVIA flattening probe is only 1 mm in diameter, which is considered to be less affected by CCT and is particularly suitable for the limited measurement of GAT (15). In this study, the high correlation and consistency of GAT and Tonopen AVIA measurements in both post-DSEK eyes and normal eyes might be due to the similar applanation technique. Of course, these results do not necessarily mean that the IOP obtained by GAT and Tonpen AVIA were absolutely accurate, because GAT is no longer a gold standard for measuring post-keratoplasty IOP.

In this study, although the GAT-IOP was the highest among all, it had no correlation with CCT, which could not indicate that the thickened cornea after DSEK made the GAT-IOP higher. Consistent with our findings, Clemmenssen and Hjortdal (13) analyzed the relationship between GAT-IOP and CCT in both FECD and DSEK eyes, and found that corneal thickening had no effect on GAT-IOP. At the same time, MacKay-Marg tonometer and Corvis ST IOP measurements were also not correlated with CCT. It is widely accepted that the biomechanical properties of the cornea have great influence on IOP measurements. As mentioned above, corneal endothelial transplantation changed host corneal structure, directly affecting corneal stiffness shown by corneal biomechanical parameters including AT1, AT2, AV1 and SP-A1. However, corneal endothelial grafts have little morphological effect on the structures that produce or outflow aqueous humor, and are therefore much less likely to in turn affect the real IOP directly. Thus, we speculate that the IOP change after DSEK may have a very small effect on corneal stiffness which affects IOP measurement. Correlation analyses showed that GAT-IOP and MacKay-Marg IOP were correlated with these biomechanical parameters, while Corvis ST IOP was not correlated with SP-A1. As for the bIOP calculated by Corvis ST which is corrected for corneal biomechanical changes, it may lead to lower IOP values in Corvis ST than in the other two tonometry systems.

This study also has some limitations. To enhance the strength of the paired comparison, we included patients with four types of typical monocular endothelial decompensation and healthy fellow eyes. Thus, this study design resulted in a rather small sample size, and was difficult to exclude confounding factors for the preoperative biomechanical properties of the different pathological corneas. In addition, this study did not evaluate the preoperative biomechanical properties of the operated eyes. As a result, changes in biomechanics and IOP both pre- and postoperatively could not be analyzed. With the innovation of Corvis ST, we are also planning to purchase the newest version and include newly released parameters for corneal stiffness such as CBI, SSI and TBI in our future studies using both Corvis ST and Pentacam.

In conclusion, the corneal biomechanical stiffness was increased after DSEK compared to normal eyes. Central corneal thickness may be less important than corneal biomechanics in measuring IOP in eyes after DSEK. The biomechanically-corrected IOP obtained by CST seems lower than other tonometry techniques in DSEK eyes, suggesting that IOP measurement with more than one tonometry may be necessary for confirming the IOP elevation.

## Data availability statement

The original contributions presented in the study are included in the article/supplementary material, further inquiries can be directed to the corresponding authors.

## Ethics statement

The studies involving humans were approved by the Ethics Committee of Eye & ENT Hospital of Fudan University. The studies were conducted in accordance with the local legislation and institutional requirements. The participants provided their written informed consent to participate in this study.

## Author contributions

HC: Conceptualization, Data curation, Funding acquisition, Investigation, Methodology, Writing – original draft. SW: Funding acquisition, Investigation, Supervision, Validation, Writing – original draft. LT: Investigation, Writing – review & editing. YL: Investigation, Writing – review & editing. JH: Supervision, Writing – review & editing. YW: Supervision, Writing – review & editing. JX: Conceptualization, Supervision, Writing – review & editing.
